# Developing an Evidence-Based Tool for Planning and Evaluating Vaccination Strategies Aimed at Improving Coverage in Elderly and At-Risk Adult Population

**DOI:** 10.3389/fpubh.2021.658979

**Published:** 2021-06-24

**Authors:** Giovanna Elisa Calabrò, Elettra Carini, Alessia Tognetto, Silvia Mancinelli, Laura Sarnari, Vittoria Colamesta, Walter Ricciardi, Chiara de Waure, The BRAVE Project Expert panel

**Affiliations:** ^1^Section of Hygiene, University Department of Life Sciences and Public Health, Università Cattolica del Sacro Cuore, Rome, Italy; ^2^V.I.H.T.A.L.I. (Value in Health Technology and Academy for Leadership & Innovation), Spin-Off of Università Cattolica del Sacro Cuore, Rome, Italy; ^3^Department of Pneumological Sciences, Section of Pneumology, University of Pavia and Fondazione IRCCS Policlinico San Matteo, Pavia, Italy; ^4^Regional Health Unit Azienda Sanitaria Unica Regionale, Area Vasta 3, Sanitary District of Macerata, Macerata, Italy; ^5^Unità Operativa Complessa Direzione Sanitaria S. Spirito e Nuovo Regina Margherita, Local Health Unit Azienda Sanitaria Locale Roma 1, Rome, Italy; ^6^Department of Medicine and Surgery, University of Perugia, Perugia, Italy

**Keywords:** vaccination, planning, evaluation, tool, vaccination strategy, vaccination coverage

## Abstract

**Background:** Vaccination coverages need to be constantly maintained and improved with the implementation of vaccination strategies. This paper describes the development of an evidence-based tool to guide their planning and evaluation.

**Methods:** A scoping review was performed in MEDLINE and institutional websites to search for similar available tools. A first version of the tool was developed considering review results and a four-step method used for the control and continuous improvement of processes and products, namely the Deming cycle. A panel of eight experts was then involved in a Delphi study for the finalization of the tool that was eventually discussed in a face-to-face meeting.

**Results:** The scoping review found only one document and the first version of the tool was composed of 30 items. After the Delphi first round, 11 additional items were suggested and 5 original items amended. After the Delphi second round 41 items were eventually included. During the face-to-face meeting, 7 items were recognized as requisites for setting vaccination strategies, whereas 17 as relevant ones.

**Conclusions:** Current public health challenges impose the need for evidence-based tools to organize effective vaccination strategies. Our tool is a first proposal which aims to reflect this focus.

## Introduction

Vaccinations are among the most relevant public health successes of the nineteenth century. They have the potential to eliminate or, at least, control an important amount of serious diseases, drastically reducing the mortality rate and complications associated with them (1). According to the latest World Health Organization (WHO) data, vaccination nowadays prevents 2–3 million deaths every year (2). In addition to reducing mortality and morbidity rates and limiting the spread of pathogens, vaccines also play an important role in the fight against Antimicrobial Resistance (3, 4).

Although the value and the benefits are recognized globally and many initiatives have been introduced to support vaccinations in recent years, in many countries a decline in vaccination coverage has been observed, with important repercussions on health and at social and economic levels (5). For example, according to the European Centre for Disease Prevention and Control (ECDC), influenza vaccination coverage among older age groups (≥65 years) in Europe (EU) during the 2015–16, 2016–17, and 2017–18 influenza seasons did not reach the EU target of 75% in any of the country that provided data and in many of them it even declined (6). In Italy, in particular, influenza vaccination coverage in elderly showed a considerably and steadily declining trend from 68.3% in the 2005–06 season to 48.6% in the 2014–15 season, turning to increase from that moment on, reaching 54.6% in the last season (2019–20) (7, 8). The same declining trend was registered for Italian vaccination coverage of Polio and Measles-Mumps-Rubella (MMR), especially in 2015 for MMR (85.3%) and in 2016 for Polio (93.3%) causing the political authorities to take measures to tackle the problem (9). The declining trend in vaccination coverages is one of the reasons why epidemics and deaths due to vaccine-preventable diseases (VPDs) still occurs also in developed countries.

The general decline in vaccination coverage can be partly attributable to the so-called vaccine hesitancy, which is known as a delay in acceptance or refusal of vaccines despite the availability of vaccine services (10). Nowadays, vaccine hesitancy represents a real threat to the health and well-being of citizens and communities, influencing the effectiveness of immunization programs all over the world. The determinants of vaccine hesitancy are various and complex and different models have been used over the time to describe the problem. The WHO Working Group on vaccine hesitancy proposed the so-called Complacency, Convenience and Confidence (3C) model. According to it, a possible reason of vaccine hesitancy might be linked to the systems that deliver the vaccination, including the competence and reliability of the own service. In fact, the decision to vaccinate is affected by the quality of the service, no matter if real or perceived, and by the level of delivery of vaccination services in terms of time and place (10). Therefore, the organization of vaccination services could be one of the underlying causes of vaccination hesitancy. In order to overcome this problem, vaccination services should be perceived as acceptable as people are more likely to vaccinate if services are convenient, easily available and without charge, if counseling is offered, and if communication and organization aspects are reasonably arranged. A proper planning of vaccination services is therefore pivotal to ameliorate vaccination attitude (1).

Also, the European Council calls the attention on the need to bring immunization services closer to citizens (11), thus, new vaccination strategies and innovative interventions are needed to promote vaccination and counter unsatisfactory vaccination coverages. Nevertheless, to our knowledge, there is not a validated tool or an institutional guideline to plan and evaluate new vaccination strategies. This paper describes the production of an evidence-based tool that is meant to help developing and appraising vaccination strategies. The tool has been built within an Italian project entitled “Best pRactices improving Vaccination coverage among at-risk adults and Elderly (BRAVE)” whose final aim was to identify and assess strategies set up in Italy in order to promote vaccination among elderly and at-risk adult population.

## Methods

The development of the evidence-based tool relied on a scoping review first and on the following application of a consensus development method, namely a Delphi. A face-to-face workshop was eventually organized in Rome on 24th September 2019 in order to discuss the final tool.

### Scoping Review and Development of the First Version of the Tool

A preliminary scoping review was performed in MEDLINE to gather evidence on tools already used and validated for planning and evaluating vaccination strategies. The following search terms were used: vaccin^*^, strategy, initiative^*^, assessment, evaluation, appraisal, grid. The search was carried out in May 2019 by two researchers independently and looked at identifying papers that proposed items for both planning and evaluating vaccination strategies in general. Additionally, other two researchers scanned the ECDC and WHO websites and gray literature. Eventual disagreements were solved by consensus.

In order to develop a first version of the tool, the four phases of the Deming cycle (planning, implementation, check, act) (12), i.e., a method used for the control and continuous improvement of processes and products were also considered as a continuous quality improvement model.

### Delphi Process

A multiprofessional panel of eight experts was involved in a 2-round Delphi survey. The Delphi survey was used to collect expert-based judgements and reach a consensus over the topic based on the assumption that a group of experts produce a more valid judgement than a single expert (13).

The experts were selected among individuals with relevant knowledge and experience of vaccination from academia (two Professors of Public Health), governmental/technical institutions (one from the Italian Ministry of Health, one from the National Institute of Health), vaccine delivery (two Directors of Public Health Departments located in tow cities of Center-Southern Italy), the public (two representatives of a non-profit citizens' organization).The two citizens' representatives participated in the survey with a single joint contribution.

The experts were contacted through email from one member of the working group who introduced the objective, the content, and the deadline of the Delphi process.

The experts were asked to express anonymously their agreement in eventually including each item of the first version of the tool through a 4-points Likert scale (“not at all,” “a little,” “quite,” “very much”). Questions were not compulsory; this choice was reasoned by the different background of experts that could have prevented them to have a clear position with respect to each item. The experts were also encouraged to propose new items and amendments to existing ones, if deemed necessary. If at least ≥70% of the experts expressed “quite” or “very much” agreement, the item was included in the final version of the tool. The cut-off of 70% was chosen arbitrarily. In the same way, if at least ≥70% of participants did not agree (“a little” or “not at all” answers) in maintaining the item, it was excluded. In the remaining cases, items were proposed again to be voted in the second round. Similarly, any amendment as well as new items proposed during the first round were submitted to experts' evaluation during the second one. In the second Delphi round, the experts voted “yes” or “no” for the inclusion of the amended and new items in the final version of the tool. As in the first round, the threshold for final inclusion was ≥70% agreement.

### Face-to-Face Workshop

The final agreement regarding the items was reached in a face-to-face meeting with the experts. Eventual disagreements were solved by discussion.

## Results

The scoping review did not yield any helpful evidence with the exception of a planning checklist for immunization campaign developed within the HProImmune project (the checklist is available at the following website: http://hproimmune.eu/toolkits/files/docs/uk/admin/planning_checklist.pdf). HProImmune (http://www.hproimmune.eu/) was a 3-year project co-funded by the Health Programme of the European Union and aimed to promote immunization among Health Care Workers in Europe. The checklist deals with the planning of immunization campaign and addresses several aspects that were also considered in the development of the first version of our tool, namely the need for a budget and for informational materials as well as the identification and evaluation of indicators.

The first version of the tool was composed of 30 items (Table 1): 13 among them regarded the planning, 7 the implementation, 4 the check and 4 the act phase. Additional two items, that did not pertain to any of the above-mentioned categories, were included among the category “other.”

**Table 1:** Items of the first version of the tool and results of the first round of Delphi.

**Phase**	**Item**	**Very much**	**Quite**
Planning	Was a working group (WG) created “*ad hoc*” for the definition and implementation of the initiative?	83%	17%
	Was the WG multi-professional and involving all possible stakeholders?	100%	
	Were patients/citizens' associations involved in the WG?	100%	
	Was the target population clearly identified?	100%	
	Was the setting *(structure)* for the implementation of the initiative identified? (4 answers).	75%	25%
	Were the health professionals responsible for the initiative identified?^a^	100%	
	Were the methods of supplying the vaccine defined?	83%	17%
	Was the project shared with the Region? (5 answers).	100%	
	Were indicators defined for evaluating the initiative?	100%	
	Were baseline vaccination coverage data collected?	100%	
	Were a protocol and a format for data collection defined?	100%	
	Was a dedicated budget defined for the initiative? (5 answers).	100%	
	Was an information action plan planned for the target population (i.e., production of brochures, posters)?	67%	33%
Implementation	Was an official launch made of the initiative?	83%	17%
	Was an active invitation made to the target population (SMS, email, telephone, social media, home letter)?	83%	17%
	Were the personnel involved in the initiative trained?	100%	
	Was the vaccination offered for free to the target population? (5 answers).	100%	
	Was pre-vaccination counseling planned and performed?	83%	17%
	Was information material distributed to the target population regarding the offered vaccination?	100%	
	Were data *(sociodemographic, adherence to the initiative, vaccine received)* on those who have joined the initiative collected? (5 answers).	80%	20%
Check	Was a final evaluation of the initiative carried out by the operators involved?	83%	17%
	Was a final evaluation of the initiative carried out by the target population *(i.e., questionnaire satisfaction)*?	83%	17%
	Was a final evaluation of the initiative conducted in terms of vaccination coverage achieved?	100%	
	Was a final evaluation of the initiative carried out in terms of costs?	83%	17%
Act	Were the results of the initiative disseminated among health professionals?	100%	
	Were the results of the initiative disseminated among the general population?	67%	33%
	Was a report produced with the final results of the initiative?	100%	
	Was any *possible* new initiative for the same objective planned based on the experience gained?^b^	66%	17%
Other	Is the initiative transferable to other contexts from an organizational point of view (resources, work group, acceptability, etc.)?	83%	17%
	Is the initiative sustainable from an economic point of view?	83%	17%

*Italics: suggested parts to be included in the item.*

a *Item moved from implementation to planning section after the first round.*

b *Item with one expert giving “A little” as answer*.

During the first round of the Delphi, there was agreement (≥70% of “quite” or “very much” answers) on all the purposed items (Table 1). All the experts did provide their answers with the exception of few items receiving four or five answers.

As a result of the first round 11 additional items were suggested (Table 2). Furthermore, 4 of the original items were amended (suggested modifications are highlighted in italics in Table 1) and 1 original item was moved from Implementation to Planning section (“Were the health professionals responsible for the initiative identified?”). During the second round all the new/amended items were accepted for the inclusion in the final tool that was eventually composed of 41 items.

**Table 2:** New items proposed for the inclusion in the tool after the first round of Delphi.

**Phase**	**Item**
Planning	Was an assessment made, at regional group level, of the type of vaccine to be used?
	Did the conservation of vaccines respected the cold chain?
	Was the project regional and/or corporate?
Implementation	Were general practitioners involved in the invitation to the initiative?
	Was a kit made available for any adverse reactions during the administration of the vaccination?
	Was consent to vaccination acquired?
	Were data collected taking into account the problems related to personal privacy?
Check	Was a final evaluation conducted in terms of the population reached by the initiative?
	Was a final assessment conducted in terms of organizational impact?
	Was a final assessment carried out with respect to the criticalities of the initiative?
Other	Is the initiative sustainable from an organizational point of view?

The face-to-face workshop served as a time for further elaborating the final tool. As a result of the discussion, primary and secondary items were identified with regard to their importance in the planning and evaluation of vaccination strategies. Furthermore, 7 items were labeled as requisites (R) that should be mandatorily met to set up a vaccination strategy. Indeed, these items were rephrased from questions to statements. The final evaluation tool was therefore amended as shown in Table 3.

**Table 3:** Final tool.

**Phase**	**Item**
Planning	**R1**. Respect of the cold chain for the conservation of vaccines.^*^
	**R2**. Identification of the health professionals responsible for the initiative.^*^
	**R3**. Definition of the methods of supplying the vaccine.^*^
	**1** Was a working group (WG) created “*ad hoc*” for the definition and implementation of the initiative?
	**1.1** Was the WG multi-professional involving all possible stakeholders?^**^
	**1.2** Were patients'/citizens' associations involved in the WG?^**^
	**2** Was the target population clearly identified?
	**3** Was the setting (structure) for the implementation of the initiative identified?
	**4** Was the project shared with the Region?
	**4.1** Was an assessment made, at regional group level, of the type of vaccine to be used?^**^
	**5** Was an information action plan planned for the target population (i.e., production of brochures, posters)?
	**6** Were a protocol and a format for data collection defined?
	**6.1** Were baseline vaccination coverage data collected?^**^
	**6.2** Were indicators defined for evaluating the initiative?^**^
	**7** Was a dedicated budget defined for the initiative?
Implementation	**R4**. Vaccination offered free of charge to the target population.^*^
	**R5**. Obtaining informed consent to vaccination.^*^
	**R6**. Collection of data taking into account personal privacy problems.^*^
	**R7**. Availability of the kit for adverse reactions during the administration of the vaccination.^*^
	**8** Were the personnel involved in the initiative trained?
	**9** Was an active invitation made to the target population (SMS, e-mail, telephone, social media, home letter)?
	**9.1** Were general practitioners involved in the invitation to the initiative?^**^
	**10** Was pre-vaccination counseling planned and performed?
	**10.1** Was information material distributed to the target population regarding the offered vaccination?^**^
	**11** Were data (sociodemographic, adherence to the initiative, vaccine received) on those who have joined the initiative collected?
	**12** Was an official launch made of the initiative?
Check	**13** Was a final evaluation of the initiative conducted?
	**13.1** In terms of vaccination coverage achieved^**^
	**13.2** Carried out by the operators involved^**^
	**13.3** Carried out by the target population (i.e., questionnaire satisfaction)^**^
	**13.4** In terms of the population reached by the initiative^**^
	**13.5** Assessment of organizational impact^**^
	**13.6** Assessment of the criticality of the initiative^**^
	**13.7** In terms of costs^**^
Act	**14** Was a report produced with the final results of the initiative?
	**14.1** Were the results of the initiative disseminated among health professionals?^**^
	**14.2** Were the results of the initiative disseminated among the general population?^**^
	**15** Was any possible new initiative for the same objective\planned based on the experience gained?
Other	**16** Is the initiative sustainable?
	**16.1** From an organizational point of view^**^
	**16.2** From an economic point of view^**^
	**17** Is the initiative transferable to other contexts from an organizational point of view (resources, work group, acceptability, etc.)?

* *Requisites.*

** *Secondary items*.

## Discussion

This paper had the aim of developing, through both the review of the available evidence and the consultation of a panel of experts, a tool for planning and evaluating vaccination strategies. The developed tool was also meant to allow the control and continuous improvement of vaccination strategies and therefore covered the four steps of the Deming cycle. The final version of the tool included items that were assigned with different relevance by the experts. Some items were actually considered as requisites, namely mandatory, for the launching and implementation of a vaccination strategy while others were considered either primary or secondary. The primary elements to consider for planning a vaccination strategy should certainly consider the actors to be involved in it (health professionals and, if possible, also representatives of citizens) and the target population to whom vaccination is directed, but also the technological, structural and economic resources necessary for the realization of the initiative. Instead, an interesting aspect deemed required for an effective implementation of the vaccination strategy was the delivery of a free of charge vaccination to the target population.

Taken as a whole, requisite addressed strategies inputs and activities laying the foundations for reaching desirable outputs and outcomes, namely regulations, human and financial resources, facilities, providers' and users' education, data collection -. It is important to point out that two requisites were those concerning the final evaluation of the strategy and the reporting of its results. These two aspects are at the center of frameworks used for program evaluation defined as a “systematic collection of information about the activities, characteristics, and outcomes of programs to make judgments about the program, improve program effectiveness, and/or inform decisions about future program development” (14). Indeed, the evaluation of health programs gained attention over the years because of the need for public health officials and health decision makers to be accountable. Nevertheless, in order to make public health strategies successful in terms of outputs and outcomes, a set of considerations should be proposed before and our tool was exactly meant to help promoting this task. The relevance of the planning phase already shines through several documents on vaccinations that anyway adopt a national viewpoint (15) or regards specific vaccinations (16, 17). Also, the COVID-19 pandemic calls the attention on the importance to plan and implement effective vaccination strategies. In particular, the European Commission (EC) pinpointed the relevance of planning adequate infrastructures and resources to make delivery and distribution of vaccines ordered, timely and fair (18). The WHO has issued a guidance to support national deployment of COVID-19 vaccines that addresses several aspects; namely, preparedness, planning and coordination, costing and funding, identification of the target population, delivery strategies, supply chain and management of health care waste, human resource management and training, vaccine acceptance and uptake, vaccine safety monitoring and management of adverse events (19). Our tool includes many of the indications suggested by the WHO but it is meant to help both developing and appraising vaccination strategies also at a more local level as it is the latter generally entrusted to deliver vaccination according to national recommendations. A study performed in Tuscany region on the implementation of maternal vaccination against pertussis highlighted meaningful factors that should be taken in mind in the planning and implementation of maternal immunization programs (20). In particular, relevant concerns emerged with respect to accountability, logistic and financial barriers, communication and training shortcomings (20). The authors also suggested some actions to deal with these issues, such as developing “an implementation plan with clear allocation of responsibilities within and across institutions” and provide educational and informational materials and training activities (20). Most of these aspects were considered in our tool. In particular, an adequate planning of activities to inform the target population is fundamental for the implementation of vaccination strategies and could help contrasting the reasons behind vaccine hesitancy (10). In fact, a more organized strategy that also includes information/communication activities could increase vaccine confidence. Similarly, a proper planning and organization of the vaccination strategy could strengthen the perceived convenience of vaccination and counteract vaccination hesitancy.

Beside these issues, also operational concerns should be addressed, namely feasibility and data tracking. These aspects were called into question in particular with respect to maternal vaccination (21, 22) and were included in our tool.

Consequently, we believe that our tool could be a useful support also in the implementation of the Immunization Agenda 2030 (IA2030) (23) that considers the vaccination as “a key contributor to people's fundamental right to the enjoyment of the highest attainable physical and mental health” envisioning a “world where everyone, everywhere, at every age, fully benefits from vaccines to improve health and well-being.” Among the strategic priorities set by the IA2030, coverage & equity goal and life-course & integration deserve a deepening as they call, respectively, for context-specific interventions and approaches to reduce missed opportunities. These two actions, in their turn, draw the attention on the need to develop and evaluate “innovative, locally tailored, evidence-based, people-centered approaches to reach poorly served populations” and to integrate vaccinations into other primary health care services (23). In our opinion, these objectives could be achieved only through a careful planning and evaluation of new vaccination strategies to be implemented at local level.

Our paper has some limitations. The most important one is that the tool was eventually built on experts' opinions as we did not find any useful evidence to develop it. Experts were all Italian, therefore items that were included in the final tool might not be applicable in other contexts, such as low-middle income countries. Furthermore, the experts were selected in an arbitrary number of eight for pragmatic issues and did not include general practitioners (GPs). Their exclusion was linked to the different level of engagement of GPs in the delivery of vaccination across the country. As for the sample size, eight participants is considered an acceptable minimum number (24); furthermore, experts involved in our study were all knowledgeable in the field of vaccination with particular regard to immunization strategies and health policies. It is important to point out that experts panel involved referents engaged in vaccination field but with various position, from academia to health management and governance and also citizens' representatives, thus allowing to have a comprehensive overview of issues that should be considered from different perspective in planning and evaluation of vaccination strategies. Anyway, the tool could be further improved with its testing in the field at national and international level. With this respect a feasibility study on its application could be helpful.

An innovative aspect of our study is represented by the application of the Deming cycle for the development of our tool. We are in fact in the context of health planning defined as a process of organizing decisions and actions to be undertaken in the present in order to achieve particular goals in the future (25). The health planning process is divided into four phases such as: conception, planning, action and evaluation (26). In this field, the use of the Deming Cycle is considered the best practice to improve the quality of systems in healthcare (12) and, for the first time, the Deming cycle has been applied to the vaccination field, providing an important methodological basis for the creation of our tool.

In conclusion, vaccinations represent a fundamental public health intervention to be supported with every mean to prevent the entire population from the burden of infectious diseases that are potentially and easily countered. Therefore, new vaccination strategies and innovative interventions are needed to promote vaccination and counter unsatisfactory vaccination coverages. To ensure this result evidence-based tools to organize effective vaccination strategies are needed. Our tool is a first proposal aimed to support this important objective.

## Data Availability Statement

The original contributions presented in the study are included in the article/supplementary material, further inquiries can be directed to the corresponding authors.

## Author Contributions

GEC, WR, and CdW conceptualized, designed, and supervised the study. AT, EC, SM, LS, and VC performed the data collection. AT, EC, SM, and LS analyzed the data and wrote the original draft of the manuscript that was revised and edited by GEC and CdW. All authors revised the manuscript and approved its submission for publication, and certifying that the work is original and their own. The expert Panel was involved in the definition of the grid.

## BRAVE Project Expert Panel

Michele Conversano, Valeria Fava, Antonio Gaudioso, Stefania Iannazzo, Roberto Ieraci, Patrizia Laurenti, Giovanni Rezza, and Stefano Vella.

## Conflict of Interest

The authors declare that the research was conducted in the absence of any commercial or financial relationships that could be construed as a potential conflict of interest.
